# Intraarticular injection of hyaluronan prevents cartilage erosion, periarticular fibrosis and mechanical allodynia and normalizes stance time in murine knee osteoarthritis

**DOI:** 10.1186/ar3286

**Published:** 2011-03-20

**Authors:** Anna Plaas, Jun Li, Julie Riesco, Rosalina Das, John D Sandy, Andrew Harrison

**Affiliations:** 1 Department of Internal Medicine (Rheumatology), Rush University Medical Center, 1735 West Harrison Street, Chicago, IL 60612, USA; 2 Department of Biochemistry, Rush University Medical Center, Chicago IL, USA; 3 Smith & Nephew Research Centre, York Science Park, Heslington, York, YO10 5DF, UK

## Abstract

**Introduction:**

Intraarticular hyaluronan (HA) is used clinically for symptomatic relief in patients with knee osteoarthritis (OA); however, the mechanism of action is unclear. In this study, we examined the effects of a single injection of HA on joint tissue pathology, mechanical allodynia and gait changes (measured by stride times) in a murine model of OA.

**Methods:**

OA was induced in the right knee joint (stifle) of 12-week-old male C57BL/6 mice by transforming growth factor β1 (TGFβ1) injection and treadmill running for 14 days. Gait parameters were quantified by using TreadScan, mechanical allodynia was evaluated with von Frey filaments, and joint pathology was evaluated by scoring of macroscopic images for both cartilage erosion and periarticular fibrosis. HA or saline control was injected 1 day after TGFβ1 injection but before the start of treadmill running.

**Results:**

OA development in this model was accompanied by significant (*P *< 0.01) enhancement of the stance and propulsion times of affected legs. HA injection (but not saline injection) blocked all gait changes and also protected joints from femoral cartilage erosion as well as tibial and femoral tissue fibrosis. Both HA injection and saline injection attenuated acute allodynia, but the HA effect was more pronounced and prolonged than the saline injection.

**Conclusions:**

We conclude that videographic gait analysis is an objective, sensitive and reproducible means of monitoring joint pathology in experimental murine OA, since stance time appears to correlate directly with OA severity. A single injection of HA prevents acute and prolonged gait changes and ameliorates the cartilage erosion and periarticular fibrosis normally seen in this model. We speculate that the capacity of HA to prevent cartilage erosion results from its normalization of joint biomechanics and its inhibitory effects on periarticular cells, which are involved in tissue hyperplasia and fibrosis. This effect of exogenous HA appears to mimic the protective effects of ablation of *Adamts5 *(a disintegrin and metalloproteinase with thrombospondin motifs 5) on experimental murine OA, and we speculate that a common mechanism is involved.

## Introduction

Progressive osteoarthritis (OA) of the knee is characterized by synovial inflammation, cartilage erosion, soft tissue fibrosis and subchondral bone sclerosis as well as pain and stiffness in the affected joints [1]. One widely used therapy for symptomatic relief of pain and stiffness is the intraarticular injection of hyaluronan (HA). A meta-analysis of clinical trials in which an aggregate total of 18 different commercial HA preparations were used summarized the results as "generally supporting the use of HA in the treatment of OA" [2] (page 3). Overall, the aforementioned analyses support the use of the HA class of products in the treatment of knee OA.

In a second meta-analysis [3], the conclusion was that HA injection led to a modest improvement in symptoms, including pain, relative to placebo. The variability in outcomes between these studies might be related to the different evaluation scales used (for example, the Western Ontario and McMaster Universities Osteoarthritis Index [4] and the Knee Injury and Osteoarthritis Outcome Score [5]) or to the finding that pain improvement with HA appears to be confined to individuals older than about 60 years of age [6].

In addition to potential symptomatic relief, there is ample evidence for statistically significant disease-modifying effects of intraarticular HA injection in both animal models and human OA [7]. For example, in rat and rabbit OA models, HA has been found to block inflammation and chondrocyte apoptosis and to prevent cartilage degeneration [8-10]. In the canine anterior cruciate ligament transection model [11], HA inhibited the formation of a fibroblast-like cell layer on the articular surface, reduced cartilage lesions and significantly improved both gross joint morphology and histopathology. In both canine and ovine OA models, subintimal fibrosis and hypervascularity of the synovium was reduced after intraarticular HA injection [12,13]. The clinical relevance of such observations is underscored by reports of human OA in which HA has been found to reconstitute the superficial cartilage layer [14], reduce synovial inflammation and edema [15] and reduce the number and aggregation of lining synoviocytes [16], as well as to reduce the progression of joint space narrowing in patients with high joint space width upon entry into the study [17]. Although the cell biological mechanisms underlying the action of HA in both animal models and human OA are poorly understood, the capacity of HA to inhibit aberrant tissue remodeling in the joint appears to be related to its efficacy in postsurgical repair in other tissues and organs. For example, ocular, thoracic and plastic surgery all involve the use of HA to optimize tissue restoration and minimize scarring [18,19], and HA is also used to prevent postoperative peritoneal and intrauterine adhesions [20,21].

The overall objective in the current work was to examine the effect of intraarticular HA injection on well-defined stages of the initiation and progression of murine OA. We have utilized a nonsurgical murine model of OA [22] which has a highly reproducible disease course leading to overt pathology in 3 weeks. In addition to performing macroscopic and microscopic evaluations of joint tissue structure, we determined mechanical allodynia (pain caused by stimuli that do not normally evoke pain [23]) and locomotive function of the hindlimbs by using the TreadScan™ gait analysis system (CleverSys Inc., Reston, VA, USA). We describe statistically robust changes in gait parameters during the different phases of OA development and show ameliorative effects of HA injection. In brief, our data show that a single injection of HA during the early transforming growth factor β1 (TGFβ1)-induced inflammatory phase of the model prevents both acute and long-term gait changes. In addition, the normalization of gait by HA injection is accompanied by a marked reduction in both cartilage erosion and intraarticular soft tissue fibrosis.

## Materials and methods

### Hyaluronan preparation

HA (Durolane; Smith & Nephew Inc., London, UK) was prepared from purified staphylococcal HA at Q-Med AB, Uppsala, Sweden. Briefly, HA is dispersed in solution and cross-linked via hydroxyl groups with butanediol diglycidyl ether. About one cross-link is formed per 100 disaccharides, and the final HA concentration is 20 μg/ml. Ten microliters of HA (75% vol/vol in saline, pH 6.8) were injected through the patellar ligament into the joint space using an insulin syringe fitted with a 30-gauge needle. The accuracy of the injection procedure for HA was verified with untreated mice (*n *= 6) using an HA/India ink solution (10:1 vol/vol) for injection, and accurate delivery was verified by the presence of the dye exclusively in the synovial cavity on dissection.

### Osteoarthritis model

Male mice (C57BL/6, age 12 weeks) were bred in-house, and all animal protocols were approved by the Rush University Medical Center Animal Care Committee. OA was induced as described previously [22]. Briefly, the procedure involves unilateral intraarticular injection of active TGFβ1 (200 ng in 0.1% (wt/vol) bovine serum albumin) at 0 and 48 hours, followed by daily enforced uphill treadmill running (17° gradient at 32 cm/second for 20 minutes) for 2 weeks.

### Experimental groups and number of mice examined

**Figure 1:**
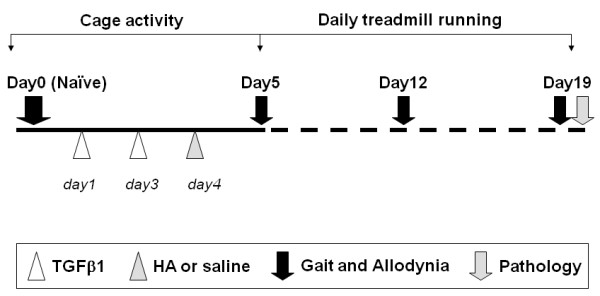
**Schematic showing the experimental protocol**. The schematic provides details of the time line used for joint injections, treadmill running and data collection. See Materials and methods for full description.

Four experimental groups were used according to the timed events illustrated in Figure 1: (1) The OA-only group (*n *= 14) received TGFβ1 injections at days 1 and 3, followed by daily treadmill running between days 5 and 19; (2) the OA + HA group (*n *= 14) received TGFβ1 injections at days 1 and 3 and an HA injection at day 4, followed by daily treadmill running between days 5 and 19; (3) the OA + saline group (*n *= 8) received TGFβ1 injections at days 1 and 3 and a saline injection at day 4, followed by daily treadmill running between days 5 and 19; and (4) the treadmill-only group (*n *= 12) were subjected to daily treadmill running between days 5 and 19.

### Gait measurement using TreadScan

Gait analysis was done using TreadScan at the time points indicated in Figure 1. The treadmill and TreadScan equipment were housed in a designated light- and temperature-controlled room. Gait measurements were always taken before treadmill running on any particular day and were carried out by the same individual throughout the study. TreadScan data on each mouse were acquired for about 20 seconds of fast walking at 18 cm/second at a 17° uphill gradient. This configuration represented optimal conditions for achieving continuous walking and more regular stride times for the C57BL/6 mouse strain used in this study. The recorded walking activity was analyzed using TreadScan version 3.0 software, which calculates the mean value for 37 locomotion parameters for each mouse from about 1,500 high-quality video frames. For the present study, data analysis was restricted to the components of the stride cycle for the hindfeet. The stride cycle represents the sum of the stance and swing times. The stance time for a hindfoot is the elapsed period between the first contact ("heel strike") and the last contact ("liftoff") with the surface and is the sum of brake and propulsion times. The brake time is the elapsed period between heel strike and the instant at which the foot reaches a position which is 80% of the distance from the front to the rear of the mouse (excluding the tail). This position represents the normal still stance position for mice. The propulsion time is the stance time minus the brake time. Presently, TreadScan does not directly evaluate loading through force plates as is done routinely in large animal studies [24] and human gait studies [25].

### Mechanical allodynia

Mechanical allodynia was determined by using von Frey filaments as described previously [23], with each 50% withdrawal threshold value (measured in grams) calculated as the average of three sequential measurements made at 5-minute intervals. Measurements were taken by the same individual for all mice and were done before treadmill running on any particular day as indicated in the schematic in Figure 1. A separate set of mice (*n *= 8) for each treatment group was used for measurement of mechanical allodynia only.

### Joint pathology

Macroscopic cartilage erosion and fibrosis scoring was done essentially as described previously [22] with the inclusion of patellar groove analysis. On day 19, right (injected) knee joints were carefully opened and menisci were removed, and after rinsing them with phosphate-buffered saline, India ink was applied to all articular surfaces with a small paint brush, and then they were rinsed again. Surfaces were photographed under a Nikon dissecting microscope (SMZ1000) (Melville, NY, USA) at sixfold magnification and images were processed using SPOT Basic imaging software (Diagnostic Instruments, Inc., Sterling Heights, MI, USA) at 32-fold magnification. For each joint, cartilage erosion scoring was carried out with the investigator blinded to sample identity. The twelve areas scored were the proximal and distal regions of the lateral and medial patellar grooves, the anterior and posterior regions of the lateral and medial femoral condyles and the anterior and posterior regions of the lateral and medial tibial plateaus. Scores (1 through 8) for each of the twelve areas were based on a visual estimate of the percentage of the specific cartilage surface affected by lesions, where a score of 8 was equivalent to 100%. Individual joint data were calculated for the patellar groove (32 maximum), the femoral condyle (32 maximum) and the tibial plateau (32 maximum) areas separately. In OA joints, macroscopic fibrosis developed at one or more of six locations. These were the posterior half of the medial and lateral tibial plateaus, the anterior half of the medial and lateral femoral condyles and the distal half of the medial and lateral patellar groove. In each location, fibrosis appeared as a variably sized whitish rim to the articular surface. Scores (0 through 3) were done with the investigator blinded to sample identity and were based on a visual estimate of the area of the lesion relative to the area (set arbitrarily at 10) of the respective articular surface. A score of 3 (maximum observed) corresponded to a lesion with an area equivalent to about 30% of the total area being scored. Individual joint fibrosis data were calculated separately for the medial and lateral anterior femoral condyles (6 maximum), the distal medial and distal lateral patellar groove (6 maximum) and the posterior lateral and medial tibial plateaux (6 maximum).

### Histology and immunohistochemistry

Following macroscopic evaluation, femoral joints were placed intact in 10% neutral buffered formalin for 48 hours, decalcified in 0.5 M ethylenediaminetetraacetic acid for 14 days, processed and embedded in paraffin. Whole joint sagittal sections (5 μm) were obtained from approximately 10 equidistant locations spanning the entire lateral and medial compartments. Sections were deparaffinized and stained with Safranin O/Fast Green. For immunohistochemistry, sections were treated with proteinase K (5 mg/ml at 37°C for 30 minutes; Sigma-Aldrich St. Louis, MO, USA) for epitope retrieval prior to primary antibody incubation. The type I collagen-specific antibody (ab34710; Abcam, Cambridge, MA, USA) was used at 10 μg/ml with incubation for 1 hour. The secondary antibody was peroxidase-coupled goat antirabbit immunoglobulin G (IgG). Preimmune rabbit IgG was used as a first antibody control.

### Data normalization and statistical analysis

Gait analyses in pilot studies using this OA model showed that a statistical power >80% can be achieved with 10 mice per group. In the study reported here, the value (expressed in milliseconds) for each gait parameter (on days 5, 12 and 19) in each hindlimb of each mouse was normalized to the corresponding pretreatment value for that mouse. This was done to minimize animal variation, which, on a weight basis, ranged from approximately 23 g to 26 g. The significance of the difference between the mean (±SD) of the normalized values and unity was determined by using an unpaired Student's *t*-test and a signed two-tailed Wilcoxon rank-sum test. The allodynia data (in grams) are the means of the 50% withdrawal threshold values for each group at each time point, and the significance of the difference between treatment groups was determined by using Student's *t*-test only. For both statistical tests, *P *values < 0.05 were considered statistically significant.

## Results

### Induction of murine OA causes an acute and prolonged increase in stance and propulsion times, and intraarticular HA injection prevents these gait changes

**Figure 2:**
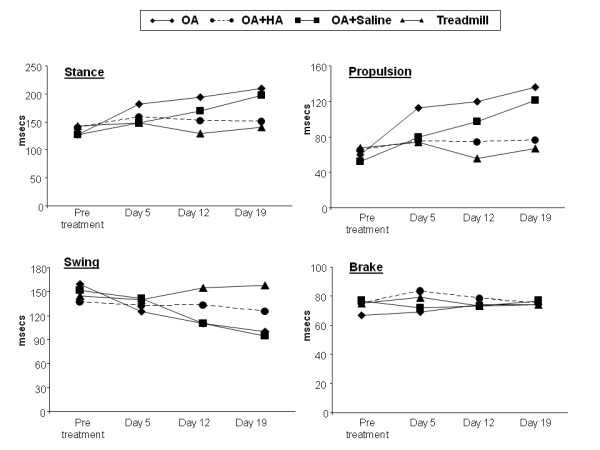
**Gait data for a representative mouse from each experimental group**. Gait data are shown in milliseconds for stance, brake, propulsion and swing times in each of the four experimental groups. Typical results from a single mouse are shown for the osteoarthritis (OA) group (diamonds), the OA + hyaluronan (HA) group (circles), the OA + saline group (squares) and the treadmill-only group (triangles).

A typical set of data (expressed in milliseconds) for stance, swing, propulsion and brake times for a representative mouse from each of the three experimental groups (OA, OA + HA and OA + saline) is shown in Figure 2. Inclusion of data for a treadmill-only mouse illustrates that there was no major change from baseline (pretreatment) values at any time. For this mouse (at all times), the percentage stance time (percentage of total stride time where stride time = stance time + swing time) was about 48% (composed of approximately 26% brake time and about 22% propulsion time), and the percentage swing time was 52%. In the OA mouse, the total stride time (stance time + swing time = approximately 280 milliseconds) was not markedly changed over the course of the experiment; however, alterations in individual percentage stance time, propulsion and swing times were detected as early as day 5, and these data were increased further on days 12 and 19. Thus, the percentage stance time increased from about 48% at baseline to approximately 75% at day 19, and this was almost entirely due to the increase in the propulsion percentage (from about 22% to approximately 47%). Accordingly, the percentage swing time decreased from approximately 53% at baseline to about 32% at day 19. The major increase in stance time (absolute value and as a percentage of stride) in the OA group was not due to a slower walking speed, since this was set at 18 cm/second on the TreadScan treadmill. Data (not shown) on stride length (expressed in centimeters) and stride frequency (expressed in Hertz) did not clearly indicate whether the OA mice changed the length and/or the frequency of strides. For the mouse from the OA + HA group (Figure 2), the total stride time was also essentially constant at approximately 280 milliseconds throughout, and there were no marked changes in individual parameters from baseline at any time. This showed that for this mouse, the HA injection had entirely prevented the OA-associated gait changes. Importantly, the effect of HA on gait was not simply due to a washout effect or a dilution of the joint fluid, since saline injection instead of HA did not block the alteration in gait parameters seen with development of OA (see OA + saline data) (Figure 2).

Next, a statistically powered study was done on the three experimental groups: for OA (*n *= 14), OA + HA (*n *= 14) and OA + saline (*n *= 8). The gait data for the affected (injected) limbs from the three groups at days 5, 12 and 19 are shown in Table 1. We present only the normalized stance and propulsion data, since in all groups and for both legs the brake time and total stride time were not statistically different from pretreatment values. Considering the means of the normalized data, it is clear that the increase over unity in stance time in the OA group (1.18, 1.40 and 1.43-fold (means ± SD) on days 5, 12 and 19, respectively) was essentially eliminated by injection of HA (1.05, 1.10 and 0.99-fold change (means ± SD) on days 5, 12 and 19, respectively). On the other hand, with injected saline the equivalent data (1.29, 1.24 and 1.32 -fold increase (means ± SD)) were not markedly altered from the OA values. The equivalent figures for the propulsion times in the OA group (1.46, 1.28 and 1.61 -fold increase (means ± SD)), the OA + HA group (1.19, 1.26 and 0.94 -fold increase (means ± SD)) and the OA + saline group (1.78, 1.16 and 1.55 -fold increase(means ± SD)) confirmed the overall inhibitory effect of HA on gait changes and the lack of an effect with saline.

**Table 1:** Effect of hyaluronan injection on the mean normalized stance and propulsion times of affected limbs in murine osteoarthritis model^a^

	Day 5			Day 12			Day 19		
Measurement	**Mean (±SD)**^**b**^	Student's *t*-test	Wilcoxon	**Mean (±SD)**^**b**^	Student's *t*-test	Wilcoxon	**Mean (±SD)**^**b**^	Student's *t*-test	Wilcoxon
Stance									
OA (*n *= 14)	1.18 (0.24)	0.009	0.014	1.40 (0.47)	0.004	0.0001	1.43 (0.40)	0.0004	0.012
OA + HA (*n *= 14)	1.05 (0.19)	0.48	0.39	1.10 (0.21)	0.19	0.12	0.99 (0.17)	0.89	0.84
OA + saline (*n *= 8)	1.29 (0.14)^c^	0.0001	N.S.	1.24 (0.26)	0.002	0.004	1.32 (0.30)	0.01	0.04
Propulsion									
OA (*n *= 14)	1.46 (0.43)	0.0005	0.002	1.28 (0.43)	0.025	0.024	1.61 (0.56)	0.0005	0.042
OA + HA (*n *= 14)	1.19 (0.31)	0.11	0.15	1.26 (0.36)	0.06	0.13	0.94 (0.36)	0.66	N.S.
OA + saline (*n *= 8)	1.78 (0.14)^c^	0.0006	N.S.	1.16 (0.43)	0.11	0.15	1.55 (0.18)	0.0001	0.008

^a^SD, standard deviation; OA, osteoarthritis; HA, hyaluronan; N.S., *P *value outside the range of Wilcoxon probability tables; ^b^data represent the means (±SD) (*n *values provided in left column) of the normalized stance and propulsion times for each experimental group on each day. The value for each gait parameter in each hindlimb of each mouse was normalized to the corresponding pretreatment value for that mouse; ^c^*n *= 4 mice for this experimental group. Values under Student's *t*-test and Wilcoxon rank-sum test headings are *P *values for the statistical significance of the difference between the means and unity.

To examine the possibility of compensatory gait changes in the contralateral limbs, we also analyzed the stance and propulsion times in the contralateral legs for the same mice (Table 2). This showed that there was essentially no statistically significant change from pretreatment values in either parameter for any treatment group on days 5, 12 and 19. The only exceptions were a reduction in propulsion time for the OA + HA group at day 5 and an increase in stance time in the OA + saline group at day 12; however, these effects were not further studied. The absence of a consistent compensatory change in the contralateral limb suggests that the increase in stance time in the affected limb is probably sufficient to stabilize rear locomotion under these test conditions.

**Table 2:** Effect of hyaluronan injection on the mean of normalized stance and propulsion times of contralateral limb in murine osteoarthritis model

	Day 5			Day 12			Day 19		
Measurement	**Mean (±SD)**^**b**^	Student's *t*-test	Wilcoxon	**Mean (±SD)**^**b**^	Student's *t*-test	Wilcoxon	**Mean (±SD)**^**b**^	Student's *t*-test	Wilcoxon
Stance									
OA (*n *= 14)	1.10 (0.34)	0.29	0.52	1.16 (0.29)	0.051	0.078	1.03 (0.13)	0.51	0.54
OA + HA (*n *= 14)	0.95 (0.15)	0.34	N.S.	1.10 (0.20)	0.15	0.080	1.10 (0.20)	0.15	0.080
OA + saline (*n *= 8)	0.97 (0.06)^c^	0.23	N.S.	1.23 (0.26)	0.026	0.046	1.21 (0.26)	0.04	0.12
Propulsion									
OA (*n *= 14)	0.92 (0.41)	0.48	N.S.	1.11 (0.42)	0.34	0.35	1.16 (0.46)	0.21	0.34
OA + HA (*n *= 14)	0.80 (0.21)	0.016	0.018	1.01 (0.19)	0.87	0.80	0.97 (0.20)	0.68	N.S.
OA + saline (*n *= 8)	0.78 (0.25)^c^	0.02	N.S.	1.05 (0.14)	0.30	0.50	1.17 (0.52)	0.36	0.69

^a^SD, standard deviation; OA, osteoarthritis; HA, hyaluronan; N.S., *P *value outside the range of Wilcoxon's probability tables; ^b^data) represent the means (±SD) (*n *values provided in left column) of the normalized stance and propulsion times for each experimental group on each day. The value for each gait parameter in each hindlimb of each mouse was normalized to the corresponding pretreatment value for that mouse; ^c^*n *= 4 mice for this experimental group. Values under Student's *t*-test and Wilcoxon rank-sum test headings are *P *values for the statistical significance of the difference between the means and unity.

### Intraarticular HA or saline reduces allodynia in OA limbs, but only HA is effective for prolonged periods

**Figure 3:**
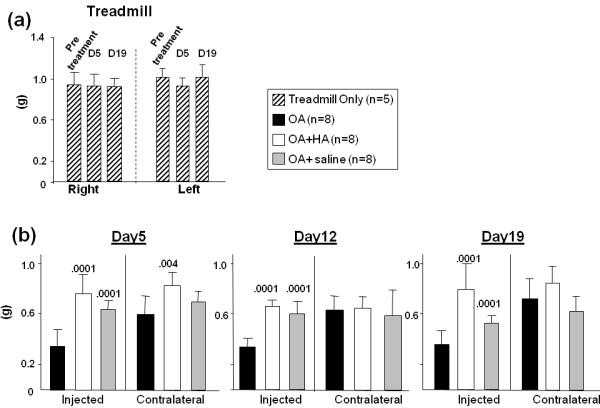
**Mechanical allodynia measurements with von Frey filaments of mice in each of the four experimental groups**. Panel (a) shows the mean 50% withdrawal threshold value (in grams) for the treadmill only mice at pretreatment, day 5 and day 19. Panel (b) shows the equivalent data for both injected and contralateral limbs (*n *= 5 to 8 per group) of mice at 5, 12 and 19 days post-injury. (see symbol box for group identification). *p *values (Student's *t*-test) are given above data sets which are statistically significantly different from the osteoarthritis (OA) group on the same day.

Since measurement of mechanical allodynia by the von Frey test has been used to monitor pain sensation during disease progression in the DMM (destabilization of the medial meniscus) model of murine OA [21], the method was also applied in this study with the nonsurgical model. The von Frey test determines a threshold score (expressed in grams) at which the mouse withdraws its paw from contact with a fine filament, so that the lower the score the more sensitive or painful is the paw or limb. Baseline analyses for untreated mice (*n *= 6) gave a mean value (±SD) of 0.95 ± 0.08 g for both hindpaws. Measurements of the treadmill-only mice taken on days 5 and 19 (Figure 3a) showed no difference from this baseline value or from the mean of their own pretreatment values, showing that treadmill running alone for up to 14 days does not generate mechanical allodynia in the hindpaws.

Measurements for allodynia in both limbs of mice from the three treated groups (OA, OA + HA and OA + saline) at days 5, 12 and 19 are shown in Figure 3b. For the OA group at day 5, the mean allodynia score in the injected limbs was approximately 0.35 g, which represents a major increase in allodynia, and this score was maintained up to day 19. The OA + HA group exhibited a significant reduction (*P *< 0.0001) in allodynia (increase in gram value) relative to the OA group at days 5, 12 and 19. The OA + saline group also exhibited a significant reduction (*P *< 0.0001) at all times, showing that for this measurement, at least part of the effect of HA appears to be due simply to the dilution of the joint fluids. However, when the same data were analyzed in terms of differences from baseline scores, the OA + HA group showed normalization (that is, the difference between mean score and mean baseline score was not significant) at days 5 and 19 and a trend to normalization on day 12. However the OA + saline group did not exhibit such normalization at any time. The greater protective effect of HA relative to saline on allodynia in the OA limb was most evident at day 19. At this time, allodynia in the OA + HA group was markedly reduced relative to the OA group (*P *< 0.0001). Allodynia for the OA + saline group was also reduced relative to the OA group, but the reduction was minor relative to the HA effect, and a return to baseline levels did not occur at any time.

Finally, the contralateral limbs of animals in the OA group and the OA + saline group showed significantly increased allodynia relative to baseline (*P *< 0.006 and *P *< 0.05, respectively) at day 5 (Figure 3b). This increase did not occur, however, in the OA + HA group, showing that at this early time, only HA had a protective effect against allodynia in both OA and contralateral limbs. In addition, the increased allodynia in the contralateral limbs was not accompanied by any change in gait parameters.

### Intraarticular HA, but not saline, protects against cartilage erosion and fibrosis in murine OA

**Figure 4:**
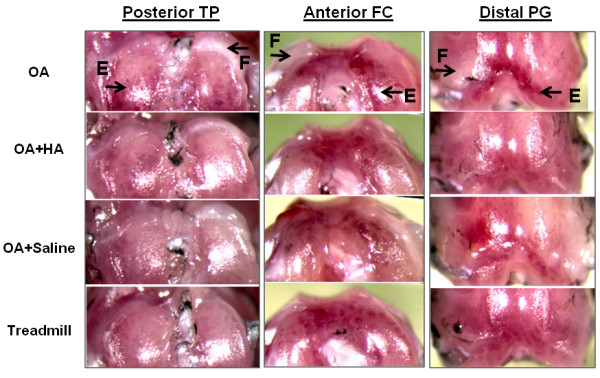
**The macroscopic appearance of cartilage and surrounding tissues from murine knee joints**. To illustrate typical cartilage erosion (E) and periarticular fibrosis (F), tibial, femoral and patellar groove images (original magnification, × 32) are shown for each of the experimental groups (osteoarthritis (OA), OA + hyaluronan (HA), OA + saline and treadmill-only) at age day 19. TP, tibial plateau; FC, femoral condyle; PG, patellar groove.

**Figure 5:**
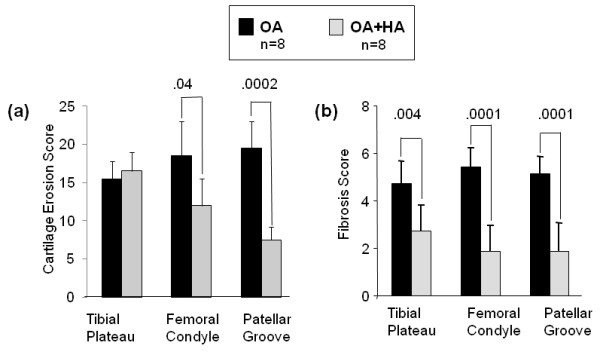
**Joint Tissue Pathology Scores showing the protective effects of prophylactic hyaluronan (HA) on murine joint osteoarthritis (OA)**. **(a) **Cartilage erosion scores and **(b) **fibrosis scores for the OA (*n *= 8) and OA + HA (*n *= 8) groups illustrates the statistically significant and area-specific effects of HA on murine OA pathology. See Materials and methods section for details on scoring.

Macroscopic evaluation of cartilage surfaces in OA joints at day 19 (Figure 4, top) showed that erosion (Figure 4, top) had progressed markedly on the posterior aspects of the lateral and medial tibial plateaus, the medial aspect of the anterior femoral condyles and the distal regions of the patellar grooves. In treadmill-only mouse joints (Figure 4, bottom), there was little if any erosion. In the OA joints, erosion sites were always adjacent to visible periarticular fibrotic deposits (Figure 4, top). Injection of HA (Figure 4, OA + HA) resulted in marked protection against cartilage erosion on the femoral and patellar groove locations, and periarticular fibrosis was diminished at all sites. In contrast, saline injection (Figure 4, OA + saline) provided only minor protection from both cartilage erosion and fibrosis. To provide quantitative comparisons, erosion and fibrosis scores were calculated for mice (*n *= 8) in each of the three groups (Figure 5). This showed that HA injection provided marked protection from femoral erosion (*P *= 0.04) but was ineffective in protecting mice from tibial plateau erosions. However, fibrotic deposits at all sites were markedly diminished in the OA + HA group (Figure 5b; *P *values shown in figure) relative to the OA group.

**Figure 6:**
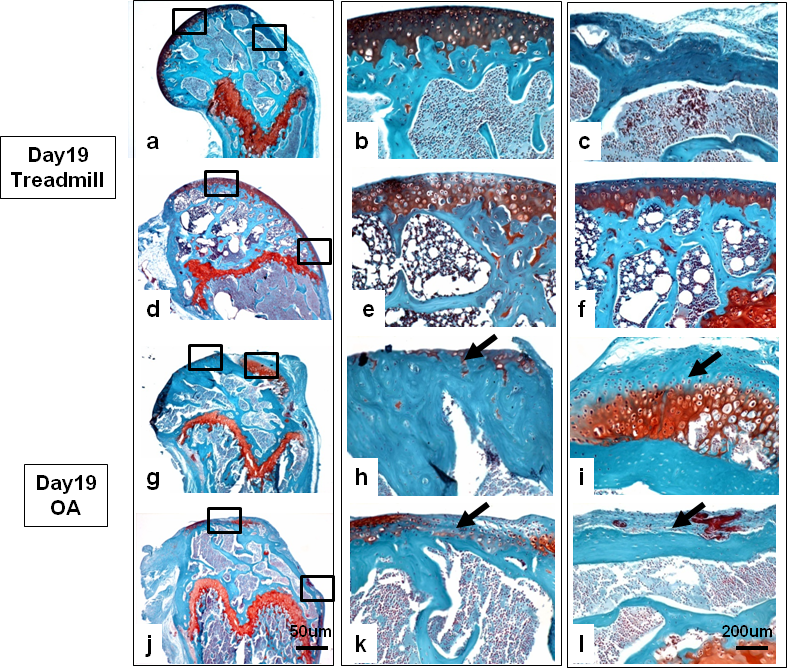
**The effect of experimental osteoarthritis (OA) on Safranin O histological staining of the murine knee joint**. Typical sections from the condyle and groove areas of **(a through f) **the treadmill-only group and **(g through l) **the OA group are shown. In each case, the boxed areas are shown at high magnification to the immediate right; for example, the boxed areas in **(a) **are shown at higher magnification in **(b) **and **(c)**. Black arrows indicate **(h and k) **cartilage erosion and **(i and l) **fibrotic or chondrophytic deposits.

**Figure 7:**
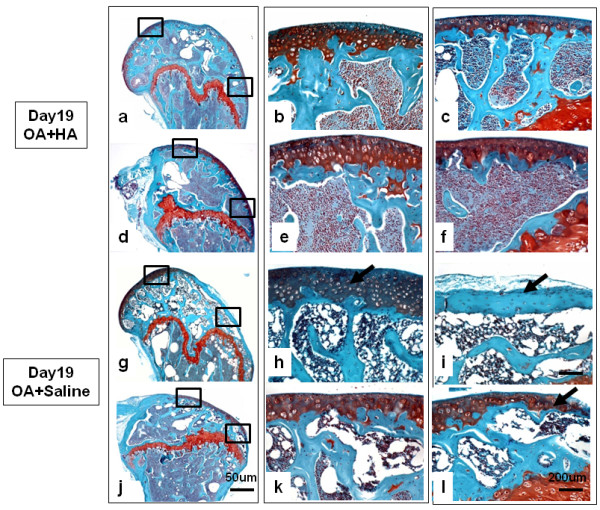
**The effect of intraarticular hyaluronan (HA) or saline on Safranin O histological staining of the osteoarthritis (OA) knee joint**. Typical sections from the condyle and groove areas of **(a through f) **the OA + HA group and **(g through l) **the OA + saline group are shown. In each case, the boxed areas are shown at high magnification to the immediate right. **(h, i and l) **Black arrows indicate cartilage loss.

To further characterize the macroscopic differences in tissue erosion and remodeling among the four groups, sagittal histologic sections from sites known to be involved (medial femoral condyle and patellar groove) were stained with Safranin O. Typical images (selected from *n *= 5 mice per group) for the treadmill-only and the OA groups are shown in Figure 6. Equivalent images for the OA + HA and OA + saline groups are shown in Figure 7. For each experimental group, two regions of the condyle and two regions of the groove are shown in both low-power fields (4-fold magnification, ) and high-power fields (20-fold magnification,). Treadmill-only femoral condyles (Figures 6a through 6f) showed intact cartilage on the central region of the condyle and the adjacent groove, as well as intact cartilage at the distal and proximal ends of the groove. OA femoral condyles (Figures 6g through 6l) had severe cartilage loss (down to the subchondral bone) on both the femoral condyle and patellar groove areas. In addition, fibrous tissue with underlying chondrophytes was detected adjacent to cartilage erosion on the anterior aspects of the medial femoral condyles (Figure 6i) and on the patellar groove (Figure 6l) in four of the five OA specimens examined. Stained sections from the OA + HA group (Figures 7a through 7f) confirmed the essentially complete cartilage protection seen macroscopically on the femoral condyle and the patellar groove. By comparison, the OA + saline group (Figures 7g through 7l) exhibited some protection against cartilage erosion and fibrous or chondrophytic remodeling in both the groove area and the posterior medial condyle, but showed no prevention of cartilage erosion on the anterior medial condyle (Figure 7l).

**Figure 8:**
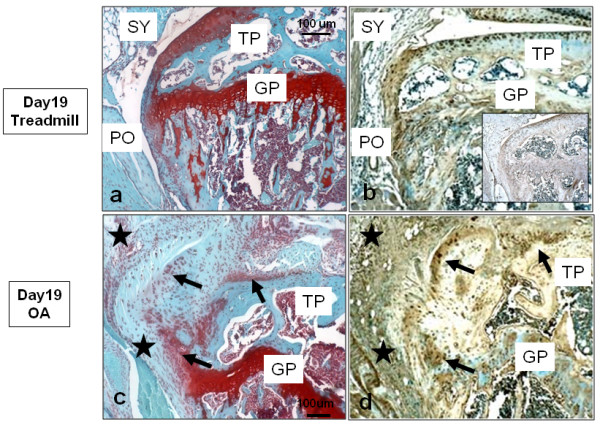
**Fibrotic tissue changes around the lateral tibial plateau associated with experimental murine osteoarthritis (OA)**. Typical sections from **(a and b) **the treadmill-only group and **(c and d) **the OA group are shown stained with **(a and c) **Safranin O or **(b and d) **antibodies to type I collagen. Black arrows indicate Safranin O-deficient tissue populated with collagen type I-positive cells **(c and d)**, respectively. PO, periosteum; GP, growth plate; TP, tibial plateau; SY, synovium.

To further illustrate the fibrotic tissue changes in the periarticular regions of the joint, sections of the lateral tibial plateaus from treadmill-only and OA joints were also stained with Safranin O (Figures 8a and 8c) or antibodies to type I collagen (Figures 8b and 8d). In treadmill-only mice, the tibial plateaus (Figure 8a) showed normal morphologies for articular and growth plate cartilages and periosteal membrane. Staining of an adjacent section for type I collagen (Figure 8b) showed it to be abundant in the cells and matrix of the periosteum. Type I collagen was also clearly detected in cells throughout the depth of the articular cartilage, illustrating the phenotypic plasticity of the chondrocyte population in 12- to 15 week-old C57Bl/6 male mice. Representative images of the OA group samples (Figures 8c and 8d) illustrate extensive loss of tibial plateau cartilage and its replacement by type I collagen-positive, Safranin O-negative fibrous ingrowths (black arrows in Figures 8c and 8d). Moreover, these ingrowths appeared to be continuous with the periosteum and synovial lining (black stars in Figures 8c and 8d). Since such fibrotic remodeling was essentially prevented by HA injection (Figures 4, 5 and 7), it seems likely that HA prevents activation of pathways that result in proliferation, migration and fibrotic differentiation of periosteal and synovial cells in this murine model of OA.

## Discussion

The data presented here (Figures 4, 5, 7 and 8) support the results of previous studies on experimental OA in rabbits and dogs showing that intraarticular HA can suppress synovial hyperplasia [10] and fibroblastic encroachment onto the articular surface [11] while protecting against cartilage erosion [26,27]. These therapeutic effects appear to be independent of the polymeric structure of HA, since the work described here was done with a cross-linked bacterial HA, whereas in other studies [8-10] non-cross-linked, high-molecular-weight preparations were found to be effective. The results of the histopathology in the current study suggest that HA blocks the ingress of activated periosteal and synovial cells and also protects against the loss of cells and matrix from femoral and patellar groove cartilages. While the precise mode of action of HA on these cells is unknown, our studies in the TTR and DMM models of OA have shown that ablation of Adamts5, prevents both cartilage erosion and fibrotic remodeling in challenged joints [20], just like HA injection in the TTR model. Since ADAMTS5 protein has been isolated in a complex with HA from both human OA cartilage [28] and degenerative equine ligaments [29], it is possible that injected HA may act primarily by blocking ADAMTS5-mediated activation of profibrotic pathways in periarticular cells.

In this regard, the current findings should provide a new framework for mechanistic studies on the effects of HA injection on OA pathology. This will be facilitated by the availability of a range of genetically modified mice in which HA-responsive components of well-established cellular pathways have been modified. For example, studies with knockout mice lacking HA-binding receptors, such as CD44 and RHAMM (receptor for hyaluronan-mediated motility) or Toll-like receptors 2 and 4, might illuminate the mechanism by which injected HA is retained in or near the joint space. Thus binding of exogenously delivered HA to such receptors might abrogate cell stress responses to joint injury, processes which might otherwise result in inflammation, cytokine release and accelerated tissue destruction [30-32].

Meta-analyses of clinical trials [3] have concluded that HA injection in humans has a real but modest effect over placebo on subjective patient pain measures. In the present work, we have used von Frey filaments to examine the potential pain-relieving effects of HA (or saline) injected during the early inflammatory phase. The most notable finding was that HA was superior to saline with regard to pain relief, but there was a marked difference only on day 19 of the model, when joint lesions were most advanced. This suggests that pain relief by HA injection includes both short-lived and longer-lived mechanisms. The short-lived effects appear to operate largely by dilution of the joint fluid, whereas the long-lived effects are probably specific to HA and may require its direct or indirect blockade of pain receptors [33]. The finding that HA, but not saline, injection eliminated the transient allodynia in the contralateral limb suggests that it may sequester factors which would otherwise enter the circulation. In this regard, Gomis *et al. *[33] suggested that HA reduces joint nociceptor activity in part because of its rheological properties but also by binding inflammatory mediators present in the joint tissues.

Probably the major contribution of the present work is the development and validation of gait analysis as an important tool in the evaluation of murine models of OA. In essence, we have shown that experimental murine OA is accompanied by no change in stride time but a consistent increase in percentage stance time (an increase from approximately 48% to about 75% in the present model) when measured at 18 cm/second on a 17° uphill gradient. Importantly, the magnitude of the percentage stance changes associated with this OA model is sufficient to readily examine the effects of therapeutic interventions. Further, the same OA mice exhibited a prolonged enhancement in mechanical allodynia in the affected limb, a readout which can also be readily monitored.

Notably, an association of OA joint damage and increased percentage stance time (at a standard velocity) was also recently reported in a study using 9-month-old type IX collagen-deficient (*Col9*^-/-^) mice [34]. The authors suggested as a possible mechanism for the increased stance time in this spontaneous OA model that the knockout mice adapted with an altered locomotion pattern (increased percentage stance time) to potentially reduce peak joint forces and thus protect the hindlimbs during walking. This idea is supported by the finding that a reduction in peak force across the normal mouse knee occurs as the total stance time increases [35].

The process that results in the increased percentage stance time in the present murine OA model remains to be investigated. An important clue may be provided by the observation that the maximum increase in percentage stance time (about 27% on day 19) was already increased by about 10% on day 5, which is before the start of treadmill running (see Figure 2 and Table 1). This indicates that the gait change can be initiated by TGFβ1 injection alone; however, it is not further increased or maintained in the absence of treadmill challenge (data not shown). It therefore may result from a loss of knee laxity due to the accompanying fibrosis of the soft tissues. Alternatively, inflammation and functional impairment in the biceps femorus, which lifts the lower hindleg during swing phase and stabilizes it during stance [36] might be a contributing factor.

Further mechanistic insight is provided by the finding in a previous study [37] that in collagen-induced inflammatory ankle arthritis, severe disease is accompanied by a decrease in stride time (due to decreases in both stance and swing times), a decrease in stride length and an increase in stride frequency (speed set at 15 cm/second). In another study [38], it was found that induction of OA by interleukin 1β injection into rat knees resulted in an asymmetric gait (measured at constant speed) in which the percentage stance time in the affected limb was reduced relative to the contralateral limb. The general finding that gait adaptations measured at constant speed are very different in ankle inflammation [37], knee inflammation [38] and knee injury (present study) illustrates the discriminatory power of gait analysis for different forms, stages and severities of murine arthritis. In this regard, the establishment of standardized procedures [39] for the evaluation of gait parameters in murine OA models should be added to existing methods, such as whole joint macroscopic pathology ([22] and present study), confocal laser-scanning microscopy [40] and histopathology [41].

Finally, it appears that the current murine study has clear clinical relevance, since human OA is also accompanied by increases in percentage stance time [42,43]. In this case, the increase results directly from the fact that during voluntary locomotion, OA patients walk more slowly than normal controls [44]. This does not, of course, exclude the possibility that OA patients have a higher percentage stance time than normal individuals at the same walking speed. In either case, it is clear that OA patients adapt to achieve walking conditions which increase percentage stance time and thereby stabilize the affected limb; however, in patients with symptomatic OA, this strategy is accompanied by higher than normal peak loading and higher than normal loading throughout stance time [45]. In this regard, the current murine model appears to accurately mimic the gait adaptations seen in human OA, although it is unknown whether the increased percentage stance time in murine OA is accompanied by higher or lower peak loading and loading throughout stance time. Whether HA therapy in human OA is accompanied by a change in walking speed and/or a reduction in percentage stance time also remains to be determined.

## Conclusions

In the present study, we have shown that a single injection of HA ameliorates both gait changes and the location-specific tissue destruction seen in the unilateral TTR murine model of OA. These findings are consistent with the conclusion that the gait change itself (that is, a major increase in percentage stance time), while apparently a protective response, may in fact be responsible for exacerbating the biomechanical and cellular biological processes which are responsible for disease progression.

We also conclude that gait analysis represents a valuable addition to the methods currently used for evaluation of the severity of joint changes in murine arthritis models. Gait analysis is particularly useful because it can provide multiple, objective, quantitative readouts on all limbs of individual mice throughout the time course of an experiment. When used in conjunction with genetically modified mice, gait analysis has a unique potential to provide mechanistic information on the relationship between locomotion and the tissue damage pathways of OA initiation and progression.

## Abbreviations

BSA: bovine serum albumin; CD44: cell surface hyaluronan receptor; HA: hyaluronan; OA: osteoarthritis; RHAMM: receptor for hyaluronan-mediated motility; TGFβ1: transforming growth factor β1.

## Competing interests

JL, JR, RD and JS declare no competing interests. AP and JL received research funding for this study from Smith & Nephew Inc. At the time of submission, AH was a full-time employee of Smith & Nephew and holds stock and shares in the company.

## Author's information

AP holds the Robert S Katz, MD-Joan and Paul Rubschlager Presidential Professorship in Osteoarthritis Research at Rush University, Chicago, IL, USA.

## Authors' contributions

AP conceived of the overall experimental design, performed intraarticular injections and blinded joint pathology scoring, and was responsible for data evaluation and interpretation and manuscript preparation. JL observed mouse treadmill running and performed TreadScan data acquisition and computation, whole joint photomicroscopy and histology, and provided draft figures and text for the manuscript. RD provided expertise in establishing von Frey methods, performed measurements and calculated data. JR performed TreadScan data acquisition and histological photomicroscopy and prepared the figures for publication. JS performed TreadScan data analyses, statistical computations and manuscript preparation. AH provided input on the overall experimental design and expertise on HA use and reviewed data interpretation with regard to published clinical studies on intraarticular HA therapy. All of the authors read and approved the final manuscript.
